# A physician-scientist preceptorship in clinical and translational research enhances training and mentorship

**DOI:** 10.1186/s12909-019-1523-0

**Published:** 2019-03-27

**Authors:** Jonathan A. Stefely, Erin Theisen, Chelsea Hanewall, Linda Scholl, Mark E. Burkard, Anna Huttenlocher, John-Paul J. Yu

**Affiliations:** 1 Medical Scientist Training Program, School of Medicine and Public Health, University of Wisconsin–Madison, Madison, WI USA; 2 Institute for Clinical and Translational Research, School of Medicine and Public Health, University of Wisconsin–Madison, Madison, WI USA; 3 Department of Medicine, Hematology/Oncology, and the UW Carbone Cancer Center, School of Medicine and Public Health, University of Wisconsin–Madison, Madison, WI USA; 4 Department of Pediatrics, School of Medicine and Public Health, University of Wisconsin–Madison, Madison, WI USA; 5 Department of Radiology, School of Medicine and Public Health, University of Wisconsin–Madison, Madison, WI USA

**Keywords:** Physician-scientist, Mentorship, Translational research, Clinical research, Curriculum, Education, Preceptorship, Apprenticeship

## Abstract

**Background:**

Dual degree program MD/PhD candidates typically train extensively in basic science research and in clinical medicine, but often receive little formal experience or mentorship in clinical and translational research.

**Methods:**

To address this educational and curricular gap, the University of Wisconsin Medical Scientist Training Program partnered with the University of Wisconsin Institute for Clinical and Translational Research to create a new physician-scientist preceptorship in clinical and translational research. This six-week apprentice-style learning experience—guided by a physician-scientist faculty mentor—integrates both clinical work and a translational research project, providing early exposure and hands-on experience with clinically oriented research and the integrated career of a physician-scientist. Five years following implementation, we retrospectively surveyed students and faculty members to determine the outcomes of this preceptorship.

**Results:**

Over five years, 38 students and 36 faculty members participated in the physician-scientist preceptorship. Based on student self-assessments (*n* = 29, response rate 76%), the course enhanced competency in conducting translational research and understanding regulation of clinical research among other skills. Mentor assessments (*n* = 17, response rate 47%) supported the value of the preceptorship in these same areas. Based on work during the preceptorship, half of the students produced a peer-reviewed publication or a meeting abstract. At least eleven peer-reviewed manuscripts were generated. The preceptorship also provided a structure for physician-scientist mentorship in the students’ clinical specialty of choice.

**Conclusion:**

The physician-scientist preceptorship provides a new curricular model to address the gap of clinical research training and provides for mentorship of physician-scientists during medical school. Future work will assess the long-term impact of this course on physician-scientist career trajectories.

**Electronic supplementary material:**

The online version of this article (10.1186/s12909-019-1523-0) contains supplementary material, which is available to authorized users.

## Background

With significant training and experience in both clinical medicine and scientific investigation, physician-scientists are well-positioned to address many contemporary challenges facing medicine [1]. The rapid pace of scientific, technologic, and even social change has placed physician-scientists at the forefront of diverse endeavors such as integrating genomics and artificial intelligence into medical practice [2, 3], improving treatments for chronic diseases such as diabetes and rare genetic disorders such as mitochondrial disease [4, 5], resolving the predicted crisis in the United States blood supply system [6], addressing the ongoing opioid epidemic [7], and developing novel cellular therapies [8]. Exciting opportunities to tackle these challenges and many others draw diverse and talented students to the field [9]. However, programs and curricula for training, developing, and retaining this critical physician-scientist workforce are not optimally designed [9–16].

Trainees encounter numerous barriers along the path towards becoming independent and productive physician-scientists, including the need to excel in both clinical medicine and scientific research, which require distinct skills, to publish high-quality work consistently, to secure funding, and to persist on this challenging path throughout an extraordinarily long training time [16]. These challenges are now amplified by increasing medical and scientific specialization, which requires extensive time for trainees to master before pursuing the goal of translational research [14]. Dual-degree MD/PhD training programs, including National Institutes of Health (NIH)-funded Medical Scientist Training Programs (MSTP), are well designed to provide rigorous training in basic science research during graduate school and in clinical practice during medical school. However, these two components of training are often largely disjointed in time and space. For example, many MD/PhD trainees spend 2–3 years in medical school, followed by 3–6 years in graduate school—often physically separated from the hospital, followed by 1–2 years in medical school prior to entering a clinical residency program. Furthermore, while training in basic science continues to be essential, the demand for clinical and translational research skills is increasing [17–20]. For example, the research landscape of physician-scientists has been dramatically impacted by the increasing availability of large electronic medical record databases that enable patient-centered research [12], but experience with approaches for using these resources is often limited. More generally, training in clinical and translational research as a whole is often limited and is a key unmet need in the field [21].

After the completion of doctoral research and medical education, aspiring physician-scientists often struggle to balance clinical work and research activities during graduate medical training and early in the first faculty appointment [22]. Following graduation and matriculation into residency programs, the focus of dual-degree trained students shifts heavily toward developing clinical skills that are required to practice medicine proficiently [23] with few provided opportunities to concomitantly pursue scientific endeavors; however, continued research experience during graduate medical education is highly beneficial. Indeed, trainees who conduct at least one year of research during graduate medical education are more likely to obtain full-time faculty positions [24]. Despite the recognized value of continuing research activities during the residency and fellowship years, structured opportunities during medical school to learn how to balance clinical and research activities are limited.

While there are inherent structural barriers to conducting research while working as a clinician, one of the primary goals of the preceptorship described here is to prepare trainees to understand this challenge and then to overcome it. One of the central means by which this can occur is by pairing trainees with senior mentors that have in practice devised strategies to balance research and clinical work. Mentor-mentee relationships established by the preceptorship can help guide junior trainees, and eventually junior faculty, through these structural challenges by relaying the experiences of senior faculty. Indeed, strong mentorship has been shown to help guide physician-scientist trainees through the above challenges [25–29], but trainees often struggle to find physician-scientist mentors, especially within their medical specialty. Students in MD/PhD programs are often mentored by PhD scientists during graduate school and by clinically-focused physicians during medical school clerkships. While some MD/PhD students work with MD/PhD physician-scientists, these mentors are often identified during the pre-clinical years before a medical specialty has been selected and are not necessarily in the students’ final specialty of choice. Formalized mentoring programs at the faculty level have long-lasting impacts on an institution’s ability to develop independent, successful scientists that have high self-efficacy and time-management scores [30, 31]. A systematic approach for connecting trainees to physician-scientist mentors within the same medical specialty could have a similarly long-lasting positive impact. To address these gaps in training and mentorship, we report herein our experience with a novel curricular advance at the terminus of MD/PhD training—the Physician Scientist Preceptorship in Clinical and Translational Research—designed to provide an opportunity for students to conduct clinical or translational research balanced with clinical practice through an apprenticeship-style learning experience with a physician-scientist mentor.

## Methods

### Setting of the preceptorship

The University of Wisconsin School of Medicine and Public Health, located in Madison, Wisconsin, United States, trains approximately 650 medical students each year, approximately 80 of whom are MD/PhD students in an NIH-funded MSTP. The Physician-Scientist Preceptorship in Clinical and Translational Research (hereafter referred to as the “preceptorship”) is a six-week clerkship offered to MD/PhD students in their final year of medical school (typically 8–10 students per year). During the first academic year in which the preceptorship was offered (2013–2014), four students had already earned the available academic credits through another clerkship, so they were ineligible to participate in the preceptorship. In subsequent years, all fourth-year medical students in the MD/PhD program were considered eligible students. The preceptorship represents a capstone experience for a set of three clerkships that are unique to the MD/PhD program at the University of Wisconsin. The other two clerkships provide opportunities for longitudinal clinical work during the graduate school research phase of the program.

### Preceptorship learning objectives and components

The core learning objectives (Table 1) and components (Table 2) of the preceptorship were designed to cover essential skills used by physician-scientists across a wide range of clinical specialties. Fundamentally, the preceptorship objectives and components are designed to help students experience six weeks in the life of a practicing physician-scientist, balancing clinical practice, research, grant writing, manuscript preparation, regulatory meetings, and public outreach—all under the guidance of a dedicated physician-scientist mentor. These course components were selected after discussing barriers to conducting translational research and common gaps in knowledge of how to conduct clinical research with our MD/PhD program directors, MD/PhD program graduates, leaders within the medical school education policy committee, and leaders within our Institute for Clinical and Translational Research. The importance of strong mentorship was emphasized and supported unanimously by these parties.

**Table 1:** Physician-scientist preceptorship learning objectives

1. Improve clinical skills and learn about the balance between clinical work and research efforts by actively participating in clinical work with a physician-scientist mentor.	
2. Engage in clinical research through an apprenticeship-style learning experience with a physician-scientist mentor.	
3. Understand clinical research design by writing a research proposal.	
4. Understand the research regulatory process, including the roles of the Institutional Review Board (IRB), Scientific Review Committee, and Protocol Review & Monitoring Committee in the regulation of clinical research by attending these meetings.	
5. Develop skills in the analysis of clinical research data.	
6. Explore the public health needs of the community and state, specifically those related to the research project, and investigate the impact of the research project on those concerns.	
7. Assess public opinion (community members and constituents) of clinical research.	
8. Develop a plan for communicating the results of the clinical research project to the public.	
9. Improve verbal and written communication skills by presenting clinical research results.

**Table 2:** Physician-scientist preceptorship components

Clinical research experience proposal	
Participation in clinical work	
Clinical research project	
Didactic lectures	
Institutional regulatory scientific meetings	
Community and health systems interviews	
Written research report	
Oral research presentation	

This preceptorship can be characterized as longitudinal [32] because research and clinical activities are integrated over the six weeks of the preceptorship, and these six weeks are preceded and followed by months of mentor-trainee interactions while developing the research project proposal and, in many cases, writing a meeting abstract or peer-reviewed manuscript. The longitudinal preceptorship format for this clerkship was selected because one of our primary aims was to enable trainees to build strong relationships with a mentor, and longitudinal preceptorships have been shown to enhance such relationships [33].

Many of the skills needed to balance research and clinical work may also be specialty-specific and even unique to a given individual, thus requiring an individualized training plan. The importance of such individualized education was recently emphasized by funding agencies requiring individualized development plans (IDPs) for trainees [34, 35]. The use of IDPs has promoted learning within the medical field and has enhanced the skills necessary for career success [36, 37]. Thus, we incorporated an individualized training program element into the preceptorship described here.

### Selection of mentors and projects

The preceptorship occurs after students have completed their core clinical rotations and have selected a clinical specialty. Through core or elective clinical rotations, students identify a physician-scientist mentor with help from the MD/PhD program directors and clerkship faculty. Once a mentor is identified, students and mentors work together to craft a project that aligns with the student’s career interests and training goals. For example, a student dedicated to cardiology with interests in learning how to use electronic medical record databases could work with a cardiology mentor to design a retrospective chart review of outcomes following admission for decompensated heart failure.

### Clinical research experience proposal

While the core course objectives and components (Tables 1 and 2) provide a general framework for the preceptorship, a more detailed plan is needed to ensure a productive clerkship for each individual student, especially given the highly customizable nature of this course. More specifically, the clinical research experience proposal is designed to: (i) formally establish the student-mentor relationship, (ii) define the student’s participation in a clinical research project, (iii) give the student an opportunity to write a concise clinical research proposal, (iv) identify opportunities for the student to participate in institutional regulatory scientific meetings, and (v) describe how the clinical research project might address one health issue listed in the state health plan. A detailed plan for each of these aspects is essential for maintaining the academic rigor of the preceptorship.

### Participation in clinical work

Participating in clinical work (seeing patients) while conducting a research project teaches students about balancing clinical time and research time as a physician-scientist. The student also learns about the patient population treated by their physician-scientist mentor. A minimum of 20 half-day clinic sessions or equivalents (e.g., four hours of radiology or pathology work time) are required with the student’s primary mentor or another attending physician in the same clinical area. To facilitate further development of clinical skills, students are expected to define and discuss clinical goals with their mentor—one of many aspects of this clerkship that are designed to help students take charge of their own learning.

### Clinical or translational research project

Active participation in clinical or translational research, guided by a physician-scientist mentor, is the primary component of this preceptorship. Students are required to participate in clinical or translational research throughout the six-week rotation. The specific nature of the research project varies by student. All projects must receive prior approval from the course director. Examples of clinical research projects are included in the Results section of this manuscript.

### Didactic lectures

Students learn about the key components of clinical and translational research through a series of required lectures. These are offered online through the University of Wisconsin School of Medicine and Public Health and its associated Institute for Clinical and Translational Research. Students must view three lectures during this course, selected from a list of options, one from each of three defined areas of emphasis: (i) research questions, (ii) study subjects, and (iii) study design.

### Institutional regulatory scientific meetings

To learn about regulation of clinical research, students attend a meeting of the Health Sciences Institutional Review Board (IRB) and a meeting of a scientific review committee (e.g., an Institute for Clinical and Translational Research Scientific Review Committee meeting or a cancer center Protocol Review & Monitoring Committee meeting). This experience is also designed to prepare students to more effectively design studies and write clinical proposals. Prior to participating in an IRB, students view an IRB workshop video online, which teaches students about when IRB review is needed, the basics of the IRB review process, what to expect at an IRB meeting, and what happens after an IRB approval or rejection. Students are required to sign a non-disclosure agreement. After participating in each institutional regulatory scientific meeting, students complete an evaluation form designed to help the student synthesize take-home points that will help them write high-quality research proposals in the future.

### Community and health systems interviews

Interviews with members of the community enable students to examine public perceptions of clinical research, what the community needs from clinical researchers, and how the student’s clinical research project might address those needs. Students are required to conduct four interviews including one interview that examines an important health issue from the perspective of a community member, one interview with a stakeholder (e.g., government official) that discusses an important health issue, and two interviews that examine clinical research from a health systems perspective (e.g., nursing staff or clinical trial coordinator). Students include a summary of each interview (400–450 words) in a written research report. The reports focus on the health issues identified by each interviewee and potential solutions for these problems.

### Written research report

The written research report consists of two parts: (i) a clinical research report and (ii) a public health impact report. The clinical research report synthesizes the results of the clinical research, ideally in a form that can be utilized by the mentor for a future publication. The public health impact report provides an opportunity for the student to act as a consultant for their research mentor on the public health impact of the research.

### Oral research presentation

The oral research presentation provides an opportunity for the student to: (i) sharpen oral presentation skills, (ii) learn how to talk about clinical research (in contrast to the basic science research conducted by most MD/PhD students during their doctoral thesis work), (iii) present the results and implications of their clinical research, and (iv) share clinical research opportunities with junior MD/PhD colleagues. This ten-minute oral presentation is given during one of the weekly sessions of the MD/PhD program seminar series attended by all members of the program.

### Survey of preceptorship and data analysis

Survey questions were developed to retrospectively evaluate whether students are meeting the objectives of the course and to help guide improvements to the preceptorship. We hypothesized that the preceptorship would increase self-perceived competency in conducting clinical or translational research. The survey questions were developed by the authors, which include the MD/PhD program architects of the preceptorship, MD/PhD program trainees, the preceptorship directors, and a member of the Institute for Clinical and Translational Research with experience in survey design. The specific questions were designed to assess changes in self-perceived competency in each of the specified preceptorship objectives (Table 1). A combination of questions with Likert-type responses and questions with free text open response options were included to enable both quantitative analysis and open-ended feedback on the preceptorship. As determined by consultation with the University of Wisconsin–Madison Institutional Review Board (IRB) and via a certification tool prior to conducting the survey, the survey was found to be exempt from a full review by the IRB due to the program evaluation and quality improvement nature of this project.

Separate surveys (Additional file 1: Figure S1, Additional file 2: Figure S2, and Additional file 3: Figure S3) were sent to all students and alumni that completed the clerkship (*n* = 38), all students in the University of Wisconsin MD/PhD program that had not yet completed the clerkship (*n* = 51) but had already selected a PhD thesis mentor, and all mentors for the clerkship (*n* = 36) (Table 3). The survey of preceptorship trainees included 17 questions, two of which were free text response (Additional file 1: Figure S1). The survey of preceptorship mentors included 11 questions, two of which were free text response (Additional file 2: Figure S2). The survey of MD/PhD students that had not yet completed the preceptorship, but had selected a doctoral thesis advisor, included three questions (Additional file 3: Figure S3). All three surveys were kept as brief as possible in order to decrease the time burden, especially for recent MD/PhD program graduates in their first or second years of residency.

**Table 3:** Preceptorship participants by academic year

Academic year	Eligible students	Preceptorship trainees	Trainee survey responses^a^	Mentors
2013–2014	7	7	7	6
2014–2015	5	4	4	4
2015–2016	10	8	5	8
2016–2017	12	10	6	10
2017–2018	10	9	6	9

^a^One survey respondent did not indicate the preceptorship year and thus is not counted in this column

We did not send surveys to the students that decided against participating in the preceptorship, primarily because we knew from previous informal discussions that some of these students opted out of the preceptorship due to personal or family hardships, and we did not want our survey to induce emotional distress for these individuals. One or two students opted out of the preceptorship in four of the five years evaluated (Table 3). The six students that opted out of the preceptorship subsequently entered a range of residency programs, including radiology, surgery, pediatrics, and family medicine, so no obvious enrichment for specialty choice was observed among these students.

In our statistical analyses, we used a non-parametric test, the Mann-Whitney test, because of the ordinal nature of the data derived from the Likert-type survey, and to avoid potentially improper assumptions about the distribution of the data. Mann-Whitney test values were determined by exact permutation (two-tailed) [38].

## Results

### Preceptorship participants and projects

The Physician-Scientist Preceptorship in Clinical and Translational Research (referred to as the “preceptorship”) was started in 2014 and continues to be conducted according to the objectives and components outlined in detail in the Methods section above and in Tables 1 and 2. During its first five years (2014–2018), 38 MD/PhD students and 36 faculty mentors participated in the preceptorship (Table 3). At least 80% of eligible MD/PhD students participated in the preceptorship each year (Table 3). The faculty members, research projects, and clinical work spanned numerous specialties across the Departments of Anesthesiology, Dermatology, Emergency Medicine, Medicine, Neurological Surgery, Neurology, Obstetrics & Gynecology, Pathology & Laboratory Medicine, Pediatrics, Psychiatry, Radiology, Surgery, and Urology. In accord with the highly customizable design of the preceptorship, a wide range of translational research projects were conducted, ranging from “Validating the MAGGIC Heart Failure Risk Model in Hospitalized Veteran Inpatients Admitted for Decompensated Heart Failure” to “Leptomeningeal Enhancement on FLAIR Imaging in Patients with Cerebral Aneurysms Treated with Endovascular Therapy”.

In 2018, to evaluate the preceptorship and to facilitate further curriculum development and potentially expansion to MD-only students, we conducted three separate surveys (Additional file 1: Figure S1, Additional file 2: Figure S2, Additional file 3: Figure S3) of three different groups: (i) trainees that completed the preceptorship, including MD/PhD program graduates, (ii) preceptorship mentors, and (iii) MD/PhD program students that had not yet taken the preceptorship, but had already selected a PhD research mentor. We received survey responses from 29 out of the 38 students who completed the preceptorship (76%) (Table 3), 39 out of 51 students who are currently in the MD/PhD program but have not yet taken the preceptorship (76%), and 17 out of 36 preceptorship mentors (47%).

Importantly, across 67 previous and current MD/PhD program students that responded to the survey question about their doctoral research classification, 58 (87%) reported conducting primarily basic science research during their PhD training, reinforcing the need for clinical and translational research training met by this preceptorship. Given the response rate of 67 (75%) out of the 89 MD/PhD program trainees and graduates eligible to answer this question, we can conclude that the majority of MD/PhD trainees in our program conduct basic science research for their doctoral thesis work. The theoretical minimum that conduct basic science research is 65%, which would only be true if all of the survey non-responders conducted clinical research. The true percentage of MD/PhD trainees that conduct basic science doctoral thesis research is more likely to be closer to the observed 87% in our sample of the population.

### Preceptorship evaluation

**Fig. 1:**
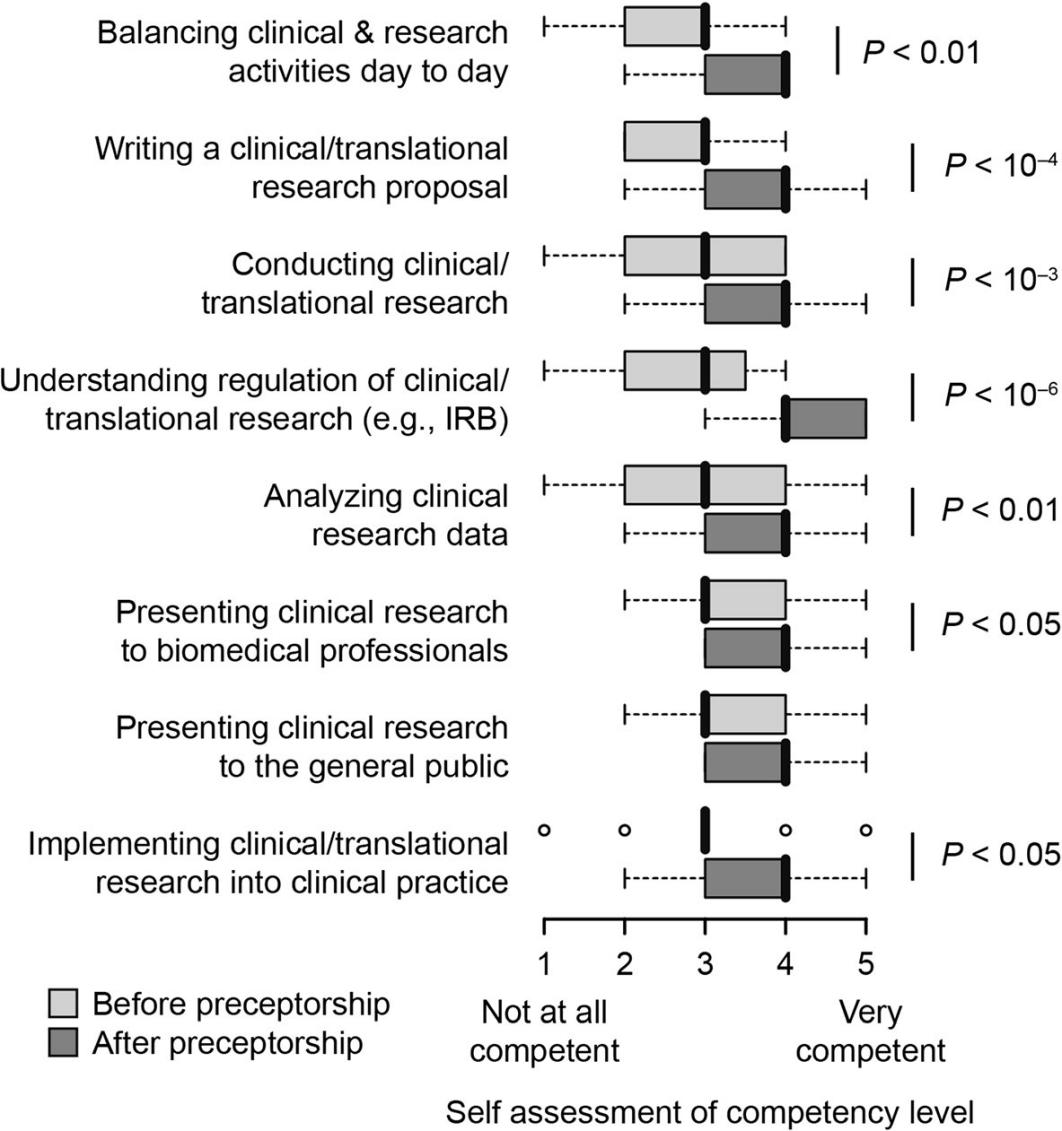
Student self-assessment of competency levels before and after taking the preceptorship. Center lines (bold) indicate medians, limits indicate 25th and 75th percentiles, whiskers extend 1.5 times the interquartile range, outliers are represented by dots, and *P* values were determined with a Mann-Whitney test (two-sided) (n = 29 respondents for both groups)

Based on student self-assessments, students felt their competency significantly increased in core skills areas including writing a translational research proposal, conducting translational research, analyzing clinical research data, balancing clinical and research activities, and implementing clinical/translational research into practice (Fig. 1). In particular student comments reflected the importance of learning how to balance clinical and research duties with statements such as “I gained some unique insight into how difficult it is to balance your clinical duties with research, especially in a surgery subspecialty,” and, “I learned that there are numerous opportunities to do clinical research in our day to day practice and that the challenge is organizing and implementing these endeavors intelligently despite our busy clinical workload.” Understanding of clinical research regulation (e.g., institutional review boards) was particularly enhanced by the preceptorship, moving most students into the confident or highly confident range (*P* < 10^− 6^). Student free response answers supported this point with comments such as “I found familiarization with the IRB process to be the most valuable part. The research was fun, the clinic was fun, the mentorship was good for networking/career building, but the area that most practically contributed to my understanding of clinical research was appreciating the review process.”

Not all competency areas were enhanced based on the survey results. The presence of specifically enhanced competencies argues against non-specific expectancy bias, which would be expected to lead to an apparent enhancement of all surveyed course learning objectives. Moreover, subgroup analyses in which the cohort was split into two groups of students—one group that completed the preceptorship recently (< 2 years) and a second group that complete the preceptorship remotely (> 2 years)—showed no significant differences in self assessments for seven of the eight skill areas, with the lone exception being an increase in self-assessed competency in conducting clinical research (*P* = 0.008) before the preceptorship among students who completed the preceptorship over two years ago (Additional file 4: Fig. S4). No difference across these two subgroups was observed for this skill after the preceptorship. This subgroup analysis suggests that recency bias was minimal in this survey.

**Fig. 2:**
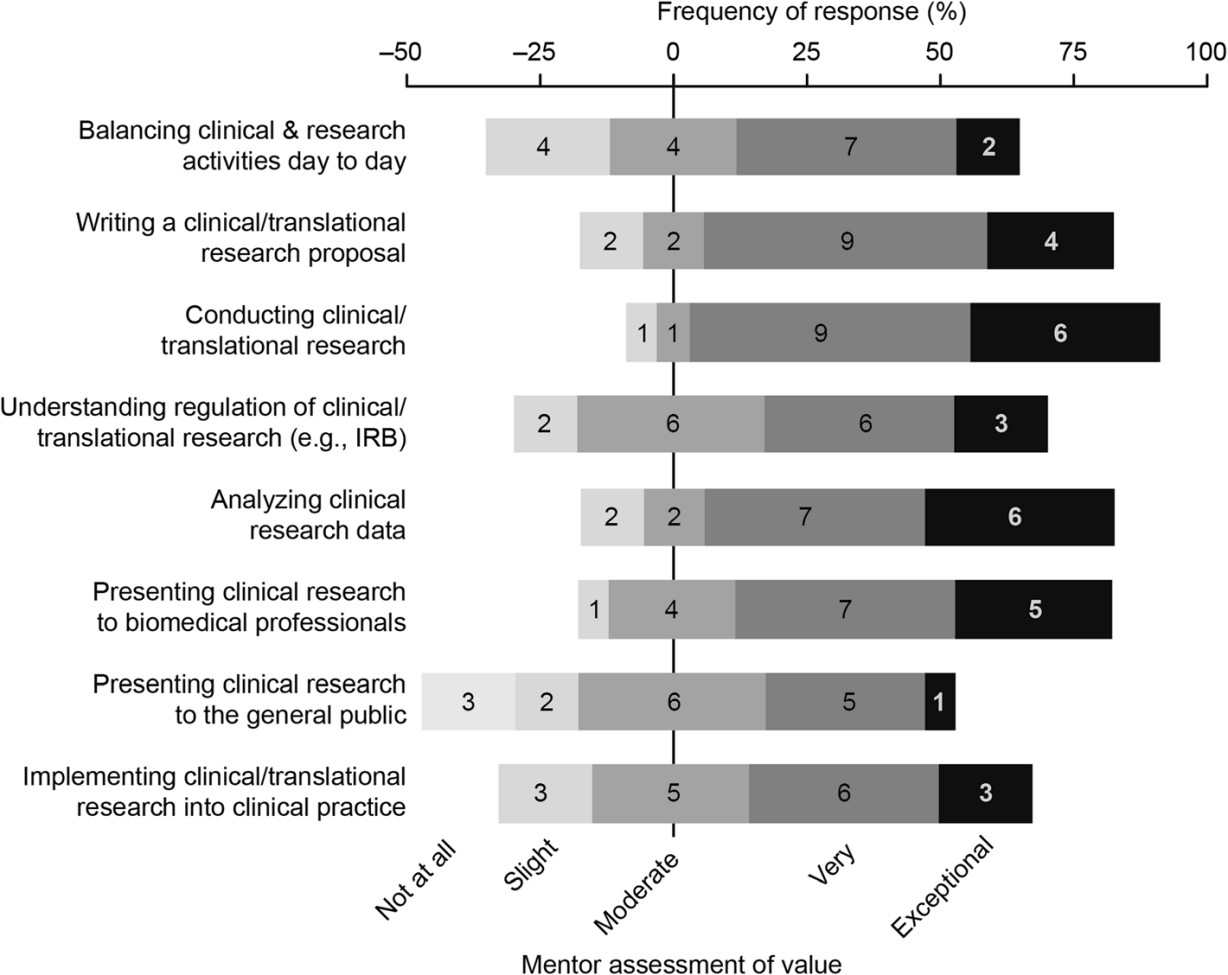
Assessment by faculty mentors that participated in the preceptorship of the value various aspects of the preceptorship (n = 17 respondents)

**Fig. 3:**
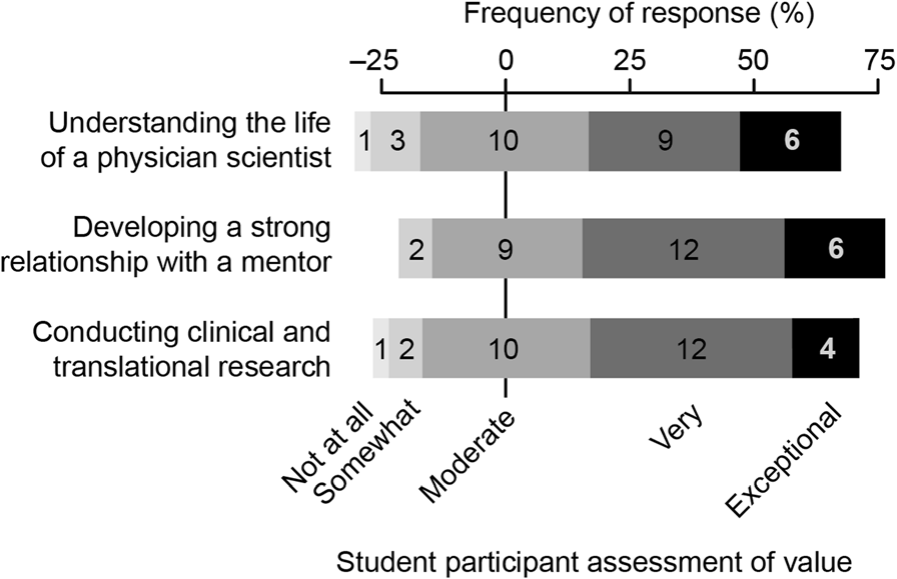
Assessment of students that participated in the preceptorship of value of the listed categories (*n* = 29 respondents)

Mentor assessments of value (Fig. 2) largely paralleled student self-assessments, particularly supporting the value of the preceptorship for experience in conducting clinical and translational research. The survey results also showed that students placed value on the development of a strong relationship with a mentor and understanding the life of a physician-scientist (Fig. 3). Free-text comments from students who completed the preceptorship also highlighted this point with comments such as, “I learned different strategies for balancing clinical practice and research time,” and “It was valuable seeing different physicians with different approaches to how they handle practical aspects of doing research, comparing and contrasting to other times in training.” Together, these results show that the preceptorship fulfills the objective of establishing a valuable mentor-mentee relationship. Competency in presenting clinical research to the general public was the only skill area that showed no significant increase after preceptorship participation based on the student self-assessment survey (Fig. 1). Concordantly, development of this skill area was suggested to be the least valuable aspect of the course by mentors (Fig. 2). Together, these findings mark development of presenting clinical research results to the general public as a weakness of the current preceptorship, for which we have recommended improvements in the discussion below.

### Scientific productivity

Based on the survey results, at least 11 peer-reviewed manuscripts based on preceptorship projects, which are distinct from the trainees’ doctoral thesis projects, were produced by the 38 students that participated in the clerkship. Some of these preceptorship-based manuscripts have already been published [39–46], while others remain under peer-review. An additional 11 abstracts were submitted and accepted for presentation at either local or national meetings, and four students identified that the data generated from their project was used in the submission of a grant application.

## Discussion

Physician-scientists continue to play important leadership roles in the biomedical research enterprise, but the pipeline for training and maintaining physician-scientists remains inefficient [9–16]. We identified a need for additional mentorship by physician-scientists in the final years of medical school training and a gap in training in clinical and translational research, especially in each student’s final clinical specialty of choice. Here, toward filling these gaps in mentorship and training, we report our experiences with a novel course, the Physician Scientist Preceptorship in Clinical and Translational Research.

Mentorship has long been recognized as essential in the development of physician-scientist leaders [25–29]. While most MD/PhD program students receive outstanding mentorship in scientific investigation by their doctoral thesis advisors, mentorship by physician-scientists within a given student’s specialty of choice is often lacking. This problem exists because during graduate school many MD/PhD students conduct basic science research mentored by PhD scientists. Moreover, many MD/PhD students change their specialty of choice between entering graduate school and applying for residency. As such, there is often a need for a new physician-scientist mentor during the final two years of the MD/PhD program. The preceptorship described here provides a structured venue for establishing a robust mentor-mentee relationship at a much deeper level than that offered by a typical clinical clerkship. For example, we have observed that potential physician-scientist mentors are often identified through a week-long interaction during a core clinical clerkship. This fleeting, yet valuable, interaction can then be continued and expanded over the next few months while planning a physician-scientist preceptorship project (as detailed in the Methods above). Subsequently, the six-week preceptorship provides further opportunities for developing a strong professional relationship, which then has the potential to be a life-long interaction. Importantly, this mentorship provides an anchor for a foundational professional network in the student’s specialty, even before entering residency.

In addition to providing specialty-specific mentorship, the preceptorship increased exposure to clinical and translational research, which has been identified as an unmet need within the training of physician-scientists [21]. More broadly, there is a general lack of translation of basic science discoveries into clinical research trials, which diminishes the impact of scientific discoveries that hold promise for major advances in human health [47]. Physician-scientists are uniquely qualified to bring basic science discoveries into the clinical realm given their extensive scientific and medical training. However, current physician-scientist training programs offer little to close the gap between basic science and the practice of medicine. Prior to participation in the preceptorship, students in our MD/PhD program had little exposure to clinical or translational research because most conduct doctoral research in basic science. Through this preceptorship, our students gained insight into bridging basic science discoveries to projects that directly impact human health, and many students feel more capable in their ability to conduct clinical research and to implement clinical research into their future practice (Figs. 1 and 3).

An additional benefit of the preceptorship is that by participating in a new research project distinct from doctoral thesis work, students expand their research network and learn how to apply previously gained skills in a new context. Recent evidence suggests that trainees are more likely to be successful in academic research if they synthesize knowledge from multiple mentors [48, 49]. The scientific productivity of the preceptorship, as demonstrated by peer-reviewed publications, abstracts, and grant submissions, suggests that trainees are gaining meaningful exposure in biomedical research fields distinct from their doctoral research. In many cases, when compared to the trainee’s doctoral research, this preceptorship research project is by design more directly related to the trainee’s future clinical specialty.

Notably, students in the preceptorship also became familiar with the regulatory aspects of clinical research such as the IRB process. The regulatory processes that are required to conduct clinical and translational research have long been identified as significant barriers that impede implementation of clinical research [50], and more recently, many clinical trials have moved out of North America to places with less regulatory burden [50, 51]. By participating in an IRB and other regulatory scientific meetings, students have the opportunity to observe the decisions made at these regulatory meetings and develop the ability to surmount barriers that slow the initiation of clinical and translational research. Though the preceptorship was largely well-received and improved students’ exposure to many aspects of clinical research, students did not gain significant exposure in presenting their work to the general public. The general public’s assessment of science is important, particularly as a basic understanding of science is critical for informed decision making [52]. To develop this skill set, we recommend adding a component to the preceptorship where students draft a press release for their research results targeted at the general public and share this with their mentors. An alternative addition could center on public engagement through social media, which has expanded since the original implementation of this preceptorship [53].

A limitation of our study is that our course was conducted at a single institution within a relatively small group of highly-motivated and independent students—those enrolled in a combined MD/PhD program. Additionally, this was a retrospective assessment of students who completed the preceptorship. The sub-group analyses discussed in detail above showed minimal differences between students who completed the course over two years ago compared to those who recently completed the course, suggesting minimal expectancy and recency bias, but we cannot definitively exclude some element of these biases. A formalized prospective assessment of students prior to participating in the preceptorship may mitigate these concerns.

## Conclusion

The physician-scientist preceptorship at the University of Wisconsin School of Medicine and Public Health has been well-received by students, expands exposure to clinical and translational research, and has been highly productive in terms of peer-reviewed manuscripts, meeting abstracts, grant submissions, and self-perceived confidence with clinical and translational research. Additional long-term benefits are anticipated because of the strong relationships developed with physician-scientist mentors and the improved understanding of the life and work of a physician-scientist. Based on the results of the first five years of our preceptorship, the experience has now been solidified as a core component of our MD/PhD program, and we expect expansion of this preceptorship to medical students outside of the MD/PhD program who plan to be physician-scientists. We anticipate that similar physician-scientist preceptorships could be successfully implemented at medical schools worldwide.

## Additional files

Additional file 1: **Figure S1.** Screen capture of the survey sent to students that completed the preceptorship. (DOCX 381 kb)

Additional file 2: **Figure S2.** Screen capture of the survey sent to mentors that participated in the preceptorship. (DOCX 188 kb)

Additional file 3: **Figure S3.** Screen capture of the survey sent to MD/PhD program students that had not yet participated in the preceptorship. (DOCX 102 kb)

Additional file 4: **Figure S4.** Student self-assessment of competency levels before and after taking the preceptorship (mean ± SD) separated by time elapsed since taking the preceptorship. *P* values were determined with a Mann-Whitney test (two-sided) (*n* = 12 for group ≤2 years; *n* = 16 for group > 2 years since taking the preceptorship). *P* values not shown were > 0.05. (DOCX 108 kb)

## Abbreviations

IRB: Institutional review board
MSTP: Medical scientist training program (a NIH-funded Dual Degree MD/PhD training program)
NIH: National institutes of health
