# Supplementary material for: A physician-scientist preceptorship in clinical and translational research enhances training and mentorship
Source: BMC Med Educ. 2019 Mar 27;19:89. doi: 10.1186/s12909-019-1523-0 (PMC6438136; doi:10.1186/s12909-019-1523-0)
Supplement: Supplementary file 1 — Figure S1. Screen capture of the survey sent to students that completed the preceptorship. (DOCX 381 kb) [file 12909_2019_1523_MOESM1_ESM.docx]

**Supplemental Figures**


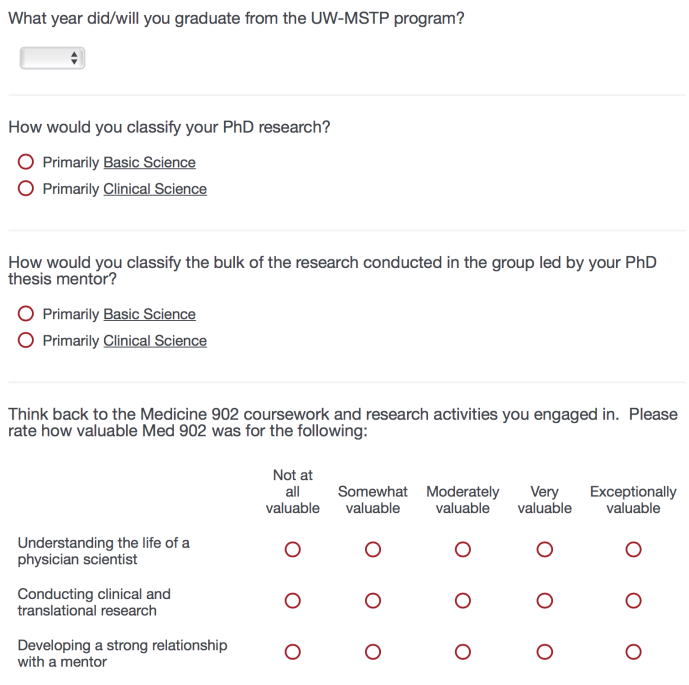

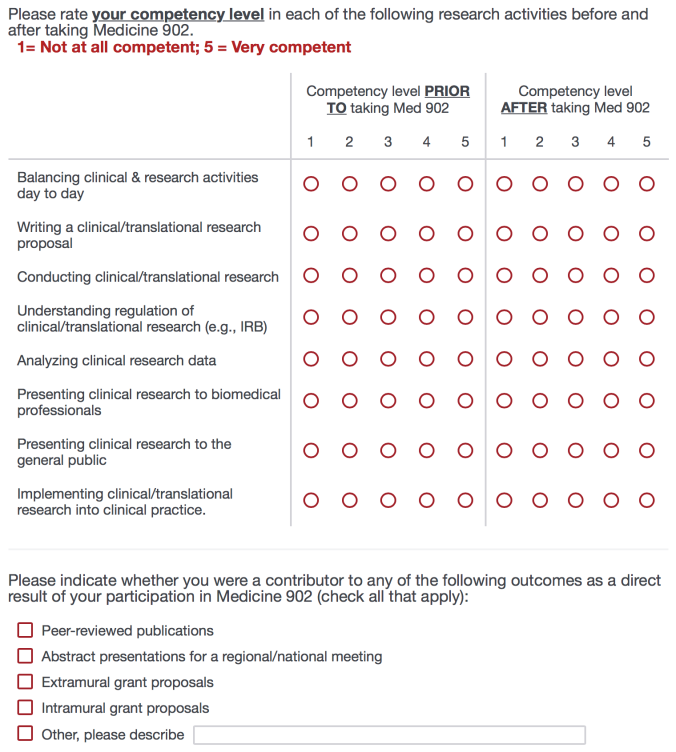


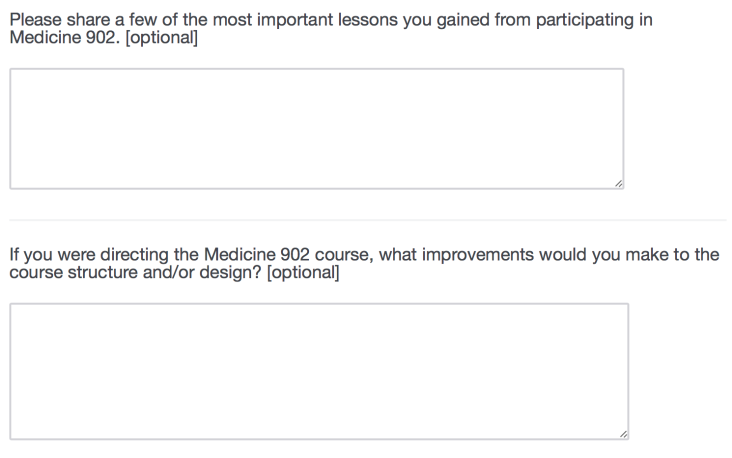


**Supplemental Figure 1**. Screen capture of the survey sent to students that completed the preceptorship.
