# Supplementary material for: A physician-scientist preceptorship in clinical and translational research enhances training and mentorship
Source: BMC Med Educ. 2019 Mar 27;19:89. doi: 10.1186/s12909-019-1523-0 (PMC6438136; doi:10.1186/s12909-019-1523-0)
Supplement: Supplementary file 3 — Figure S3. Screen capture of the survey sent to MD/PhD program students that had not yet participated in the preceptorship. (DOCX 102 kb) [file 12909_2019_1523_MOESM3_ESM.docx]

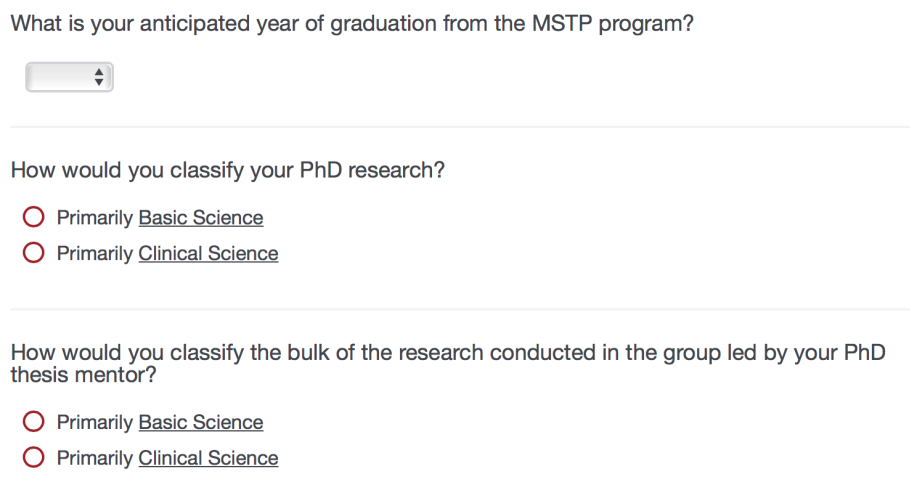


**Supplemental Figure 3**. Screen capture of the survey sent to MD/PhD program students that had not yet participated in the preceptorship.
