# Supplementary material for: A physician-scientist preceptorship in clinical and translational research enhances training and mentorship
Source: BMC Med Educ. 2019 Mar 27;19:89. doi: 10.1186/s12909-019-1523-0 (PMC6438136; doi:10.1186/s12909-019-1523-0)
Supplement: Supplementary file 4 — Figure S4. Student self-assessment of competency levels before and after taking the preceptorship (mean ± SD) separated by time elapsed since taking the preceptorship. P values were determined with a Mann-Whitney test (two-sided) (n = 12 for group ≤2 years; n = 16 for group > 2 years since taking the preceptorship). P values not shown were > 0.05. (DOCX 108 kb) [file 12909_2019_1523_MOESM4_ESM.docx]

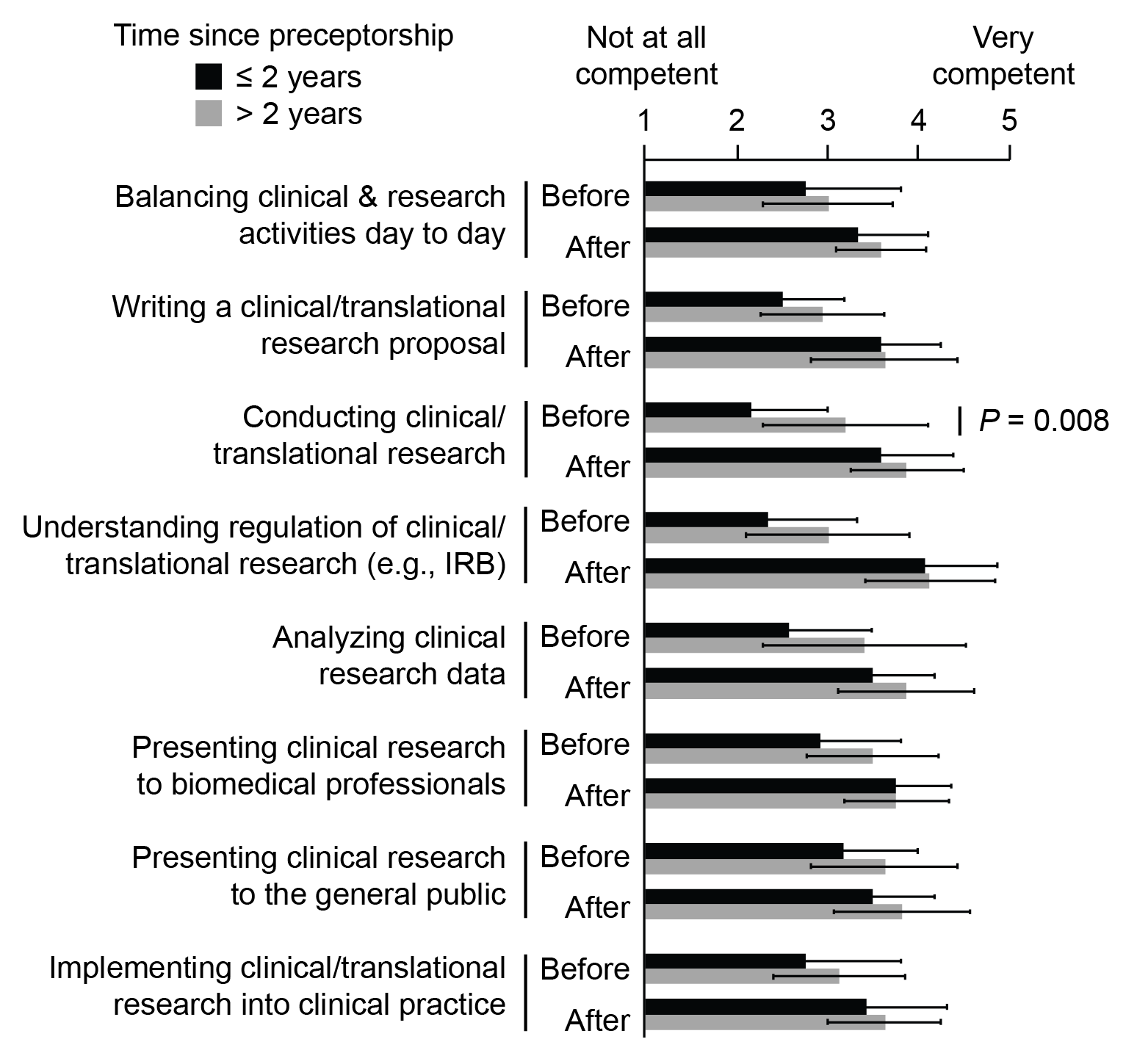


**Supplemental Figure 4**. Student self-assessment of competency levels before and after taking the preceptorship (mean ± SD) separated by time elapsed since taking the preceptorship. *P* values were determined with a Mann-Whitney test (two-sided) (n = 12 for group ≤ 2 years; n = 16 for group > 2 years since taking the preceptorship). *P* values not shown were > 0.05.
